# Medium-Chain Triglycerides Lower Blood Lipids and Body Weight in Streptozotocin-Induced Type 2 Diabetes Rats

**DOI:** 10.3390/nu10080963

**Published:** 2018-07-26

**Authors:** Ming-Hua Sung, Fang-Hsuean Liao, Yi-Wen Chien

**Affiliations:** 1 School of Nutrition and Health Sciences, Taipei Medical University, Taipei 11031, Taiwan; Glycine1016@hotmail.com (M.-H.S.); function0727@gmail.com (F.-H.L.); 2 Research Center of Geriatric Nutrition, College of Nutrition, Taipei Medical University, Taipei 11031, Taiwan; 3 Graduate Institute of Metabolism and Obesity Sciences, Taipei Medical University, Taipei 11031, Taiwan

**Keywords:** type 2 diabetes mellitus, medium-chain triglyceride, long-chain triglyceride, lipid metabolism

## Abstract

Medium-chain triglycerides (MCTs) are distinguished from other triglycerides in that each fat molecule consists of 6 to 12 carbons in length. MCTs and long-chain triglycerides (LCTs) are absorbed and utilized in different ways. The aim of this study was to assess the effects of replacing soybean oil with MCT oil, in a low- or high-fat diet, on lipid metabolism in rats with streptozotocin-induced type 2 diabetes mellitus (T2DM). There were, thirty-two T2DM Sprague-Dawley rats divided into low-fat-soybean oil (LS), low-fat-MCT oil (LM), high-fat-soybean oil (HS), and high-fat-MCT oil (HM) groups. After 8 weeks, blood sugar, serum lipids, liver lipids, and enzyme activities related to lipid metabolism were measured. Under a high-fat diet condition, replacement of soybean oil with MCT oil lowered serum low-density lipoprotein cholesterol (LDL-C), non-esterified fatty acids, and liver total cholesterol; whilst it increased serum high-density lipoprotein cholesterol (HDL-C) and the HDL-C/LDL-C ratio. A low-fat diet with MCT oil resulted in lower body weight and reproductive white adipose tissues compared to the HS groups, and higher hepatic acyl-CoA oxidase activities (the key enzyme in the peroxisomal beta-oxidation) compared to the LS group in T2DM rats. In conclusion, MCTs showed more protective effects on cardiovascular health in T2DM rats fed a high-fat diet, by improving serum lipid profiles and reducing hepatic total cholesterol.

## 1. Introduction

Type 2 diabetes mellitus (T2DM) is the predominant form of diabetes worldwide and is accompanied with a heavy economic burden [1]. From a pathophysiological standpoint, persons with T2DM consistently show three cardinal abnormalities: (1) resistance to the action of insulin in peripheral tissues particularly muscles and fat; (2) defective insulin secretion, particularly in response to a glucose stimulus; and (3) increased glucose production by the liver. The pathogenesis of T2DM is complex and involves diet imbalances, life stresses, and obesity [2].

The prevalence rates of being overweight and obese in Taiwan, defined by the Taiwanese definition (body-mass index = 24~26.99 and ≥27 kg/m^2^, respectively), were 22.9% and 10.5% for men and 20.3% and 13.2% for women, respectively [3]. Obesity reduces life expectancy and increases the risks of several non-communicable diseases (i.e., T2DM, hypertension, and dyslipidemia).

Medium-chain triglycerides (MCTs) are distinguished from other triglycerides in that each fat molecule is between 6 and 12 carbons in length. Medium-chain fatty acids (MCFAs) are transported in the portal blood directly to the liver, unlike low-chain fatty acids (LCFAs), which are incorporated into chylomicrons and transported through lymph [4]. Moreover, MCFAs do not require a carnitine shuttle system to penetrate mitochondria [5]. In β-oxidation, MCFAs cause the greatest induction of medium-chain acyl coenzyme A (CoA) dehydrogenase and increase the oxidation rate. Overall, the MCT transport pathway and the oxidation rate, differ from those of long-chain triglycerides (LCTs) [6]. The weight gain of rats fed an MCT-containing diet was 30% less than that of rats fed an LCT-containing diet [7]. MCTs have a reductive effect on body fat stores [8]. Body weight was significantly reduced (17%) in weanling rats fed high-MCT diets [9]. The decreased deposition of fat in MCT-overfed rats may result from obligatory oxidation of MCT-derived fatty acids in the liver [10]. MCT may decrease fat accumulation in adipocytes by increasing thermogenesis and satiety [11]. MCTs also improved plasma triglyceride and total cholesterol concentrations in rats [12].

Previous research, has verified that MCT oil has acceptable effects on body weight, fat, and blood lipids [7,8,9,10]. The aim of this study was to assess the effects of replacing soybean oil with MCT oil, in a low- or high-fat diet, on lipid metabolism and enzyme activities in rats with T2DM. The study had two hypotheses: (1) that MCTs improve cardiovascular health in T2DM, shown by loss of body weight and fat; and (2) that MCTs reduce serum and liver lipids accompanied by regulation of liver lipid metabolic enzyme activities.

## 2. Materials and Methods 

MCT oil (lot no. 1959) was obtained from Kao Corporation (Tokyo, Japan). The fatty acid component percentages of the MCT and soybean oils were analyzed via gas chromatography [13]. The medium-chain fatty acid (MCFA) and long-chain fatty acid (LCFA) proportions of the MCT oil were 87.1% (containing 32.1 ± 0.5% caprylic acids, 37.9 ± 0.2% capric acids, and 17.2 ± 0.1% lauric acid) and 12.9%, respectively. There were no MCFAs in the soybean oil.

### 2.1. Animals and Diets

Male Sprague-Dawley rats (*n* = 60; aged 5 weeks with a body weight (BW) of 126~150 g) were obtained from Lasco (Taipei, Taiwan). Rats were housed individually in wire-bottomed stainless-steel cages, in an air-conditioned room (21 ± 2 °C and 50~70% relative humidity) with a 12-h light: dark cycle and free access to a basic diet and water for 1 week before diabetes was induced. Diabetes was induced via a high-fat diet (58% of calories as fat) for a period of 2 weeks, and an intraperitoneal injection of a low dose of streptozotocin (STZ) (35 mg/kg). After 2 weeks, a rat was considered diabetic when its fasting blood glucose concentration was ≥180 mg/dL [14]. At this point, baseline blood samples were collected from the tail vein of rats after anesthetization with ether gas. We then began to feed the rats with the experimental diets. Thirty-two T2DM Sprague-Dawley rats were divided into 4 groups, with similar average initial body weights. T2DM rats were divided into low-fat with soybean oil (LS), low-fat with MCT oil (LM), high-fat with soybean oil (HS), and high-fat with MCT oil (HM) groups. The LS diet per kilogram contained 70 g soybean oil (16% of calories as fat); the LM diet contained 35 g soybean oil (8% of calories as fat) and 38 g MCT oil (8% of calories as fat); the HS diet contained 254.4 g soybean oil (58% of calories as fat); and the HM diet contained 127.2 g soybean oil (29% of calories as fat) and 137.9 g MCT oil (29% of calories as fat). All diets contained 200 g casein and 50 g a-cellulose as fiber/kg of diet. Choline, cysteine, minerals, and vitamins were added as described in AIN-93 [15]. Calorie densities of low-fat diets and high-fat diets were 4 kcal/g and 5.1 kcal/g, respectively. The weight of animal diets was adjusted to fix total caloric intake and all rats received 109 kcal/day for 8 weeks. Calories from the daily food intake, during the experimental period did not differ among the groups (data not shown). After consuming the diets for 8 weeks, the rats were deprived of food overnight (~14 h); then they were anesthetized with ester. Blood was centrifuged at 3500 *g* at 4 °C for 10 min, and serum was collected. The livers and reproductive white adipose tissue (RDWAT) were removed. All samples were frozen at −80 °C until being analyzed. All animal experimental procedures followed published guidelines and were approved by the Institutional Animal Care and Use Committee of Taipei Medical University (Taipei, Taiwan).

### 2.2. Intraperitoneal Glucose Tolerance Test (IPGTT)

At 0 and 8 weeks, all rats were fasted for approximately 16 h and intraperitoneally injected with 50% glucose solution (0.1 mL/100 g), and venous blood samples were obtained at 0, 15, 30, 60, 90, 120 and 180 min to determine plasma glucose.

### 2.3. Assay of Serum and Hepatic Lipids

Blood glucose, triglyceride, total cholesterol (TC), high-density lipoprotein cholesterol (HDL-C), low-density lipoprotein cholesterol (LDL-C), and non-esterified fatty acid (NEFA) concentrations were determined spectrophotometrically using kits from Randox (Taipei, Taiwan). The serum insulin concentration was measured with a rat insulin enzyme-linked immunosorbent assay (ELISA) kit (Mercodia, Taipei, Taiwan). Triglycerides, cholesterol, and NEFAs in liver samples were extracted [16], and levels were measured using kits from Randox.

### 2.4. Enzyme Assay

The fatty acid synthase (FAS) activity assay, was based on measuring the initial rates of total NADP+ formation from NADPH, by cytosomes by an ELISA. The cytosomes in the medium were incubated with 2 M potassium phosphate, 20 mM dithiothreitol, 0.25 mM acetyl-CoA, 60 mM EDTA-2Na, and 0.39 mM malonyl-CoA, and the reaction was monitored at 340 nm [17,18].

The acyl-CoA oxidase (ACO) activity assay, was based on the method of Small et al. by an ELISA reader [19]. Liver postnuclear supernatant medium, were taken at 1% of total reaction volume and incubated with 11 mM potassium phosphate, 8 μg/μL horseradish peroxidase type II, and the 5% LAT mixture (contained 2.5 mM DCFH-DA, 4 M aminotriazole, and 20% Triton X-100). After 6 min (30 °C), 3 mM palmitoyl CoA was added. The reaction was monitored at 502 nm every minute for a total of 10 min, and the rate of H_2_O_2_ formation was calculated.

The acetyl-CoA carboxylase (ACC) activity assay [20], was based on measuring the initial rates of total NADP+ formation from NADPH, by cytosomes by an ELISA. Cytosomes in the medium, were incubated with 20 mM Tris-HCl, 10 mM MgCl_2_, 10 mM potassium citrate, 3.75 mM glutathione, 12.5 mM KHCO3, 0.675 mM bovine serum albumin (BSA), 0.125 mM acetyl-CoA, and 3.75 mM ATP, and the reaction was monitored at 340 nm.

The carnitine palmitoyltransferase (CPT) activity assay [21], was based on measuring the initial rates of total free CoA (CoASH) formed by the 5,5′-dithio-bis-2-nitrobenzoic acid (DTNB) reaction from palmitoyl CoA by individual mitochondria with l(-) carnitine by an ELISA. Mitochondria in the medium, were incubated with 116 mM Tris-HCl buffer, 0.09% Triton X-100, 1.1 mM EDTA-2Na, 0.035 mM palmitoyl CoA (Sigma, Darmstadt, Germany), 0.12 mM DTNB, and 1.1 mM 1(-) carnitine. The reaction was monitored at 412 nm.

The assay of 3-hydroxy-3-methylglutaryl coenzyme A (HMG-CoA) reductase activity was based on measuring the initial rates of total NADP+ formed from NADPH, by microsomes by an ELISA. Microsomes in the medium, were incubated with 0.2 M KCl, 0.16 M potassium phosphate, 0.004 M EDTA, 0.01 M DL-dithiothreitol, 0.1 mM HMG-CoA, and 0.2 mM NADPH, and the reaction was monitored at 340 nm [22,23,24].

### 2.5. Histology

Livers and RDWATs were steeped in 10% formalin, dehydrated, and packed in wax. After 2 days, the specimens were stained with a hematoxylin and eosin solution.

### 2.6. Statistical Analysis

All data are expressed as the mean ± SEM. Data from multiple groups were compared by two-way ANOVA, and each group was compared with the others by Fisher’s protected least significant difference (LSD) test. Statistical significance was defined as *p* < 0.05.

## 3. Results

### 3.1. Weight Gain and Organ Weights

Calories in the daily food intake during the experimental period did not differ among the groups. After 8 weeks of treatment, the average body weight in the HS group was higher than in the other three groups, and it was significantly higher than that of the LM group (*p* < 0.05, see Table 1). The average RDWAT (%) in the HS group was significantly higher than that of the LM group (*p* < 0.05). The average liver weight (*w*/*w*) in the HS group was lower than in the other three groups, and it was significantly lower than that of the LM group (*p* < 0.05). The average brown fat and kidney weights of rats did not differ among the groups (Table 1).

### 3.2. Blood Glucose, Insulin Concentrations, Intraperitoneal Glucose Tolerance Test (IPGTT) and Lipid Levels

**Figure 1:**
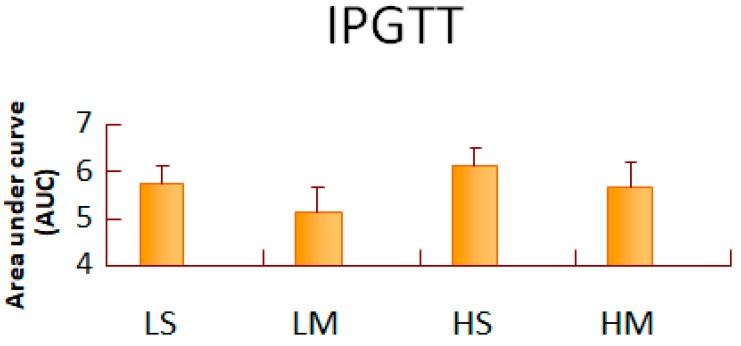
Area under the curve (AUC) of intraperitoneal glucose tolerance test (IPGTT) of type 2 diabetes mellitus rats fed the different diets for 8 weeks. Data are expressed as mean ± SEM (*n* = 8). LS: diet contains 16% soybean oil, LM: diet contains 8% soybean oil and 8% medium-chain triglyceride oil, HS: diet contains 58% soybean oil, HM: diet contains 29% soybean oil and 29% medium-chain triglyceride oil. All values are multiplied by 10^−4^.

**Figure 2:**
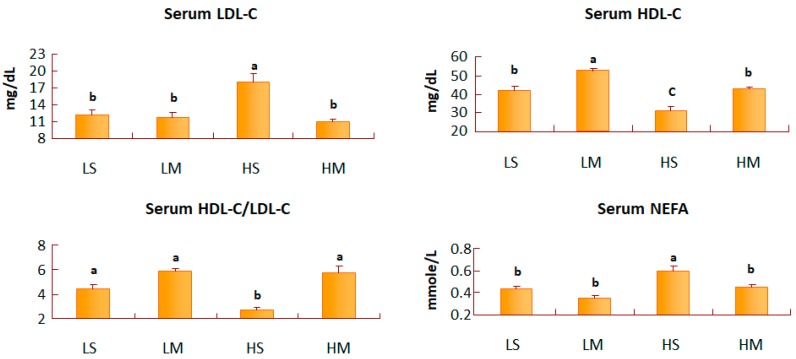
Serum low-density lipoprotein cholesterol (LDL-C), high-density lipoprotein cholesterol (HDL-C), the HDL-C/LDL-C ratio, and non-esterified fatty acid (NEFA) concentrations of type 2 diabetes mellitus rats fed the different diets for 8 weeks. Data are expressed as the mean ± SEM (*n* = 8). LS, diet contained 16% soybean oil; LM, diet contained 8% soybean oil and 8% medium-chain triglyceride oil; HS, diet contained 58% soybean oil; HM, diet contained 29% soybean oil and 29% medium-chain triglyceride oil. Values with different superscripts significantly differ at *p* < 0.05.

After 8 weeks of treatment, blood glucose and serum insulin concentrations of T2DM rats did not differ among the four groups (Table 2). The LM group had the least area under the curve (AUC) of IPGTT, and the HS group had the greatest AUC of IPGTT (Figure 1). The LM group had a lower TG concentration than the HS group (*p* = 0.07) (Table 2). The blood TC concentration did not differ among the groups. The serum LDL-C concentration of the HS group was significantly higher than in the other three groups (*p* < 0.05), and that in the HS group was higher than that of the HM group alone (*p* < 0.05). The HS group had a lower HDL-C concentration than the HM group. Also, the MCT oil groups had significantly higher HDL-C values than the soybean oil groups, with both the low- and high-fat diets. The serum HDL-C/LDL-C ratio of the HM group was significantly higher than that in the HS group (*p* < 0.05), and the NEFA concentration of the HS group was significantly higher than that of the HM group (*p* < 0.05) (Figure 2).

### 3.3. Liver Lipid Levels

**Figure 3:**
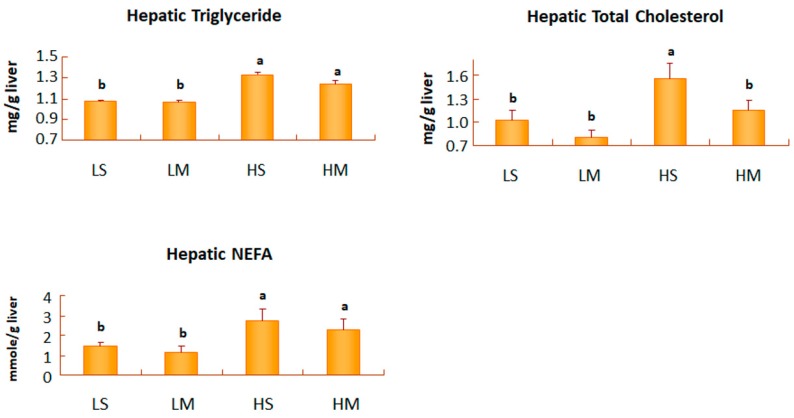
Hepatic triglyceride, total cholesterol, and non-esterified fatty acid concentrations of type 2 diabetes mellitus rats fed the different diets for 8 weeks. Data are expressed as the mean ± SEM (*n* = 8 in each group). LS, diet contained 16% soybean oil; LM, diet contained 8% soybean oil and 8% medium-chain triglyceride oil; HS, diet contained 58% soybean oil; HM, diet contained 29% soybean oil and 29% medium-chain triglyceride oil. Values with different superscripts significantly differ at *p* < 0.05.

After 8 weeks of treatment, the low-fat diet groups had significantly lower hepatic TG levels, than the high-fat diet groups (*p* < 0.05, Figure 3). The HS group had the highest levels of the hepatic TG, TC, and NEFA. In groups with a low-fat diet, the hepatic TG, TC, and NEFA levels of the MCT oil and soybean oil groups did not significantly differ. However, the hepatic TC levels of the HM group, were significantly lower than those of the HS group (*p* < 0.05, Figure 3).

### 3.4. Liver Enzyme Activity Assay

**Figure 4:**
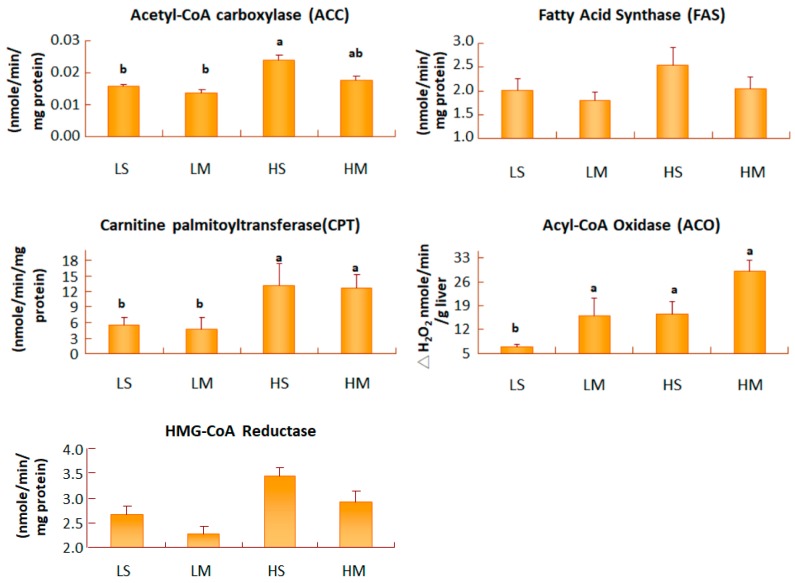
Liver acetyl-CoA carboxylase, fatty acid synthase, acyl-CoA oxidase, carnitine palmityltransferase, and HMG-CoA reductase activities of type 2 diabetes mellitus rats fed the different diets for 8 weeks. Data are expressed as the mean ± SEM (*n* = 8 in each group). LS, diet contained 16% soybean oil; LM, diet contained 8% soybean oil and 8% medium-chain triglyceride oil; HS, diet contained 58% soybean oil; HM, diet contained 29% soybean oil and 29% medium-chain triglyceride oil. Values with different superscripts significantly differ at *p* < 0.05.

Hepatic FAS is the enzyme involved in de novo lipogenesis pathway, leading to the accumulation of fatty acids in tissues. After 8 weeks of treatment, no difference in hepatic FAS activity was found among the four groups (Figure 4). The formation of malonyl-CoA from acetyl-CoA was an irreversible process, catalyzed by ACC. T2DM rats, fed both low-fat diet groups had significantly lower hepatic ACC activities than those fed the HS group (*p* < 0.05). There were no differences in hepatic ACC activity between the HM group and the LM group. ACO is the key enzyme in the peroxisomal β-oxidation pathway. When the activity of ACO increases, β-oxidation increases. In T2DM rats fed low-fat diets, the MCT oil group had higher activity of hepatic ACO increases than the soybean oil group (*p* < 0.05). Liver carnitine palmitoyltransferase (CPT), is the key step in mitochondrial β-oxidation for long-chain fatty acid, binding to carnitine from cytosol to the matrix. T2DM rats fed a low-fat diet had significantly lower hepatic CPT activities than the high-fat diet groups (*p* < 0.05). HMG-CoA reductase, is the key enzyme for endogenous cholesterol synthesis in the liver. In groups with a low-fat diet, the MCT group had slightly less activity than the soybean oil group; the same result was found for the high-fat diet (Figure 4).

### 3.5. Histology

**Figure 5:**
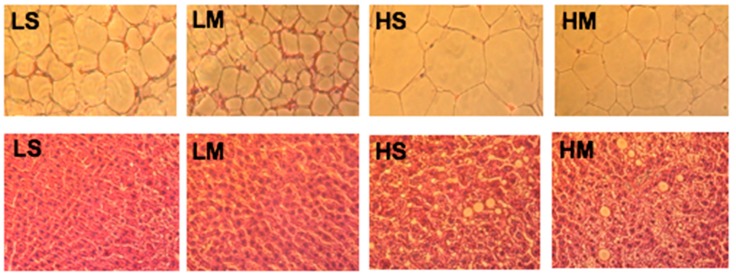
Reproductive white adipose tissues (upper row) and liver tissues (lower row) of type 2 diabetes mellitus rats fed the different diets for 8 weeks, shown with hematoxylin and eosin staining (10×). LS, diet contained 16% soybean oil; LM, diet contained 8% soybean oil and 8% medium-chain triglyceride oil; HS, diet contained 58% soybean oil; HM, diet contained 29% soybean oil and 29% medium-chain triglyceride oil.

Examination of RDWAT slices showed that the HS group had the largest adipocytes, and the LM group had the smallest (Figure 5). The LM group had smaller adipocytes than the LS group; the same result was found in high-fat diet groups. The HM group had smaller adipocytes than the HS group. The liver tissue slice results showed that the two high-fat diet groups had more lipid droplets than the two low-fat diet groups, corresponding to the hepatic TG concentrations (Figure 5).

## 4. Discussion

After 8 weeks of treatment, the body weight of diabetic rats consuming a high-fat diet with soybean oil was significantly higher than that of rats consuming a low-fat diet with MCT oil (*p* < 0.05). MCT undergoes faster and more-complete hydrolysis to MCFA than LCT to LCFA [25,26], and MCFAs are absorbed and oxidized more quickly than LCFAs, reducing body weight by decreasing fat accumulation [4,5,6]. However, the daily calories from food intake during the experimental period did not differ among the groups. Additionally, the body weight of the LS group was approximately 18% less than that of the HS group. Thus, the LM group compared with the HS group, not only had MCT oil, but also had a lower percentage of oil in the diet. The LM group had a significant decrease in RDWAT compared to the HS group. MCT reduces the percentage of body fat by increasing the activity of hepatic lipolytic enzymes (ACO) [6]. Thus, feeding a low-fat with MCTs-rich diet, caused a much greater reduction of body weight and adipose tissues in T2DM rats.

In the low-fat diet, the MCT oil group had a slightly smaller AUC (glucose) than the soybean oil groups; the same was found for the high-fat diet. Insulin resistance in mice is reduced by a low-fat diet [27]. Also, the area under the curve can be an index of the body’s insulin resistance [28]. MCT oil consumption might have benefits to blood glucose levels and insulin resistance [27], although our results did not have statistical significance. In our study, the serum TG of the LM group was less than that of the HS group, and the serum NEFA concentration in the HM group was significantly lower than that of the HS group. MCT oil reduced hepatic TG synthesis by decreasing hepatic lipogenic enzyme (ACC) activity, increasing hepatic lipolytic enzyme (ACO) activity, and lowering serum TG [6]. Reducing the percentage of oil in the diet may have the same effect [29]. Thus, MCT reduced body fat more effectively with a low-fat diet. The serum LDL-C concentration of the HS group was significantly higher than that of the HM group. The number of hepatic LDL receptors is reduced significantly by high insulin concentrations [30,31]. High hepatic TC also diminishes hepatic LDL receptors [32]. Thus, MCT oil might increase the recovery of serum LDL-C, accompanied with the reduction in serum NEFA concentrations, by increasing hepatic LDL receptors, not hepatic HMG-CoA reductase activity. The most common form of atherosclerosis in diabetes, is induced by abnormal LDL-C metabolism [33]; rats in the HS group were most likely at risk of atherosclerosis. In addition, subjects with higher plasma small-dense LDL levels have up to a 3-fold increased risk of myocardial infarction [34]. Future research, may need to investigate the effect of MCT oil on advanced lipoprotein particles. The serum HDL-C concentrations of the MCT oil groups were significantly higher than those of the soybean oil groups, in both the low- and high-fat diets. Defective ABCA1 mediating the efflux of cellular free cholesterol, defective LCAT activity, or increased selective delivery of HDL cholesteryl ester to hepatocytes may be involved in the low HDL levels present with severe insulin resistance [35]. The serum HDL-C/LDL-C ratio of the HM group was significantly higher than that of the HS group. The HDL-C/LDL-C ratio can be a predictor of the risk of coronary heart disease [36]. Thus, the HS group might have the highest risk of coronary heart disease. Additionally, MCTs enhanced serum HDL-C concentrations, and may have achieved more efficient protection of cardiovascular health in T2DM rats fed a high-fat diet.

After 8 weeks of treatment, the low-fat diet groups had significantly lower hepatic TG and NEFA levels than the high-fat diet groups. A high-fat diet induced TG accumulation in adipose tissues. In T2DM, resistance to the action of insulin in adipose tissues increased NEFAs from TG hydrolysis and released them to the blood, and seriously high NEFAs in the blood were carried into the liver where the de novo synthesis of TGs occurred [37]. Hepatic TC levels in the HM group were significantly lower than those of the HS group; but hepatic HMG-CoA reductase activity in the HM group did not differ compared to that of the HS group. Takase et al. reported that hepatic HMG-CoA reductase activity was significantly lower in MCT-fed rats [38]. The reason why a high-fat with MCT oil diet reduced hepatic TC levels, accompanied with higher serum HDL-C levels, might be associated with hepatic LDL receptors [32] and other proteins, such as the hepatic scavenger receptor (SR-B1) and hepatic cholesterol 7-hydroxylase (CYP7A1), involved in liver cholesterol metabolism [39,40].

White adipose tissue is an energy storage and is related with insulin resistance of T2DM and cardiovascular complications of obesity. With a low-fat diet, replacement of soybean oil with MCT oil may improve fat oxidation by increasing hepatic ACO activities to reduce reproductive white adipocyte size. In some animal studies, the mean adipocyte size was smaller in MCT- than in LCT-fed rats [10]; those of the LM group were smallest. In obese and diabetic B6 mice that were switched from a high- to a low-fat diet, obesity was completely reversed [41]. Thus, the small adipocyte size of our LM group was related to consumption of MCT, accompanied with a reduction in the percentage of oil. Rats fed a higher-fat diet had higher ACC and FAS activities, and a faster hepatic lipogenetic pathway to accumulate TGs in the liver. The liver tissue slice results corresponded to hepatic TG levels.

## 5. Conclusions

Consequently, we concluded that MCTs can achieve more efficient protection of cardiovascular health in T2DM rats fed a high-fat diet, by improving serum lipid profiles and reducing hepatic total cholesterol. Moreover, T2DM rats fed a low-fat and MCTs-rich diet, had much greater losses of body weight and adipose tissues, compared with those fed a high-fat with soybean oil diet.

## Figures and Tables

**Table 1:** Body and organ weights of type 2 diabetes mellitus rats fed the different diets for 8 weeks.

	LS	LM	HS	HM
0 week-Weight (g)	353.8 ± 11.5	354.1 ± 8.0	357.4 ± 18.7	350.6 ± 12.81
8 week-Weight (g)	373.1 ± 18.4 ^ab^	340.3 ± 14.3 ^b^	435.4 ± 12.0 ^a^	392.8 ± 19.2 ^ab^
RDWAT (g)	5.1 ± 1.0	4.9 ± 0.9	9.3 ± 0.9	8.4 ± 1.2
RDWAT %	1.2 ± 0.2 ^ab^	1.2 ± 0.2 ^b^	2.1 ± 0.2 ^a^	1.9 ± 0.2 ^ab^
BAT (g)	0.3 ± 0.1	0.3 ± 0.0	0.33 ± 0.0	0.34 ± 0.0
BAT %	0.1 ± 0.0	0.1 ± 0.0	0.1 ± 0.0	0.1 ± 0.0
Liver (g)	11.5 ± 0.6	12.5 ± 0.6	12.6 ± 0.4	12.1 ± 0.4
Liver %	3.2 ± 0.1 ^ab^	3.4 ± 0.1 ^a^	3.0 ± 0.1 ^b^	3.1 ± 0.1 ^ab^
Kidney (g)	3.0 ± 0.1	3.2 ± 0.1	3.7 ± 0.2	3.1 ± 0.1

Data are expressed as mean ± SEM (*n* = 8 in each group). LS, diet contained 16% soybean oil; LM, diet contained 8% soybean oil and 8% medium-chain triglyceride oil; HS, diet contained 58% soybean oil; HM, diet contained 29% soybean oil and 29% medium-chain triglyceride oil. RDWAT, reproductive white adipose tissue; BAT, brown adipose tissue. Values with different superscripts significantly differ at *p* < 0.05.

**Table 2:** Blood glucose, insulin, triglyceride, and total cholesterol concentrations of type 2 diabetes mellitus rats fed the different diets for 8 weeks.

	LS	LM	HS	HM
Blood glucose (mg/dL)	302.5 ± 15.4	287.9 ± 22.7	368.2 ± 32.8	297.9 ± 22.1
Insulin (ug/L)	0.4 ± 0.1	0.4 ± 0.1	0.5 ± 0.1	0.4 ± 0.1
Triglyceride (mg/dL)	55.9 ± 2.0	52.2 ± 1.8	69.2 ± 5.0	57.4 ± 3.6
Total cholesterol (mg/dL)	63.8 ± 3.3	63.3 ± 3.6	70.6 ± 1.6	64.2 ± 2.7

Data are expressed as the mean ± SEM (*n* = 8 in each group). LS, diet contained 16% soybean oil; LM, diet contained 8% soybean oil and 8% medium-chain triglyceride oil; HS, diet contained 58% soybean oil; HM, diet contained 29% soybean oil and 29% medium-chain triglyceride oil.
